# Cholera—Its Protean Aspects and Its Management

**Published:** 1893-07

**Authors:** 


CHOLERA, ITS PROTEAN ASPECTS AND ITS MANAGEMENT, by Dr. G. A. Stockwell, F. Z. S., in two volumes. Detroit, Mich. Geo. S. Davis, 1893.

These two little volumes, of "The Physician's Leisure Library," are of more than passing interest; first, because of the possibility, not to say probability, of cholera getting a foothold in American seaports during the present year; and, secondly, because the author has had the courage to pronounce against the theories of Koch and his followers. Dr. Stockwell believes cholera to be the result of profound poisoning, produced by some agent allied to the toxic alkaloids and acting directly upon the central nervous system.

